# Exploring the Potential of Macroalgae for Sustainable Crop Production in Agriculture

**DOI:** 10.3390/life14101263

**Published:** 2024-10-03

**Authors:** Domenico Prisa, Roberto Fresco, Aftab Jamal, Muhammad Farhan Saeed, Damiano Spagnuolo

**Affiliations:** 1 CREA Research Centre for Vegetable and Ornamental Crops, Via Dei Fiori 8, 51012 Pescia, Italy; 2 CREA Research Centre for Engineering and Agri-Food Transformation, Council for Agricultural Research and Economics, Via della Pascolare 16, 00016 Monterotondo, Italy; roberto.fresco@crea.gov.it; 3 Department of Soil and Environmental Sciences, Faculty of Crop Production Sciences, The University of Agriculture, Peshawar 25130, Pakistan; 4 Department of Environmental Sciences, COMSATS University Islamabad, Vehari Campus, Vehari 61100, Pakistan; farhansaeed@cuivehari.edu.pk; 5 Department of Chemical, Biological, Pharmaceutical and Environmental Sciences, University of Messina, Salita Sperone 31, 98166 Messina, Italy; damiano.spagnuolo@unime.it

**Keywords:** sustainable agriculture, biofertilizers, marine macroalgae, seaweed extracts, bioactive compounds, plant protection

## Abstract

Marine macroalgae, which typically colonize coastal areas, are simple plant organisms. They live on rocks in coastal regions and are classified into red, brown, and green macroalgae. These algae are an important natural resource in agriculture due to their ability to enhance the structural, chemical, and biological properties of soil. Marine macroalgae can be used to produce various biocidal molecules that are effective in controlling plant pathogens. Much of the literature on marine macroalgae and their derivatives focuses primarily on the pharmaceutical field, while their use in agriculture is still considered secondary. However, various studies and experiments have demonstrated their potential to play a significant role in crop protection and enhancement. This review aims to highlight the various applications of macroalgae in plant production. It also emphasizes the biotechnological importance of marine macroalgae derivatives as biofertilizers, molecules for controlling insects and microorganisms, and as plant growth conditioners. Compounds from macroalgae, such as fatty acids, carotenoids, polyphenols, and carbohydrates, are being investigated for their fungicidal, antimicrobial, and antiviral effects against various plant pathogens. Beyond enhancing crop production, macroalgae can also be considered multifunctional bioinoculants suitable for use in organic farming.

## 1. Introduction

Plant disease management is a crucial aspect of sustainable agriculture [1]. Although pesticides have traditionally been used to protect crops and maintain high yields, their use can lead to various health and environmental problems [2]. In the early 2000s, insecticides made up approximately 30% of the global pesticide market, herbicides 50%, fungicides and bactericides 25%, and other products 4% [3,4,5]. Some of the main environmental concerns relate to the persistence of pesticides in soils and their leaching into groundwater, but also to the undesirable effects they can have on non-target organisms in general [6,7,8]. However, products of natural origin are considered less hazardous due to their greater biodegradability and use at lower doses [9]. Natural products are also of interest in the development of new biological products for pest control, as they provide the raw material for safer crop protection products [10]. For centuries, humans have exploited the sea for the production of economically important substances [11]. Over the past 50 years, many marine organisms have been isolated and characterized. While various macroalgae inhabit the seas and oceans, some species can also colonize freshwater ecosystems [12,13].

Macroalgae are divided into three groups: red algae (*Rhodophyta*), green algae (*Chlorophyta*), and brown algae (*Phaeophyta*). Green algae have the same green color as plants due to chlorophylls *a* and *b*. Red algae have a red color due to the main pigment phycoerythrin. Brown algae are brown due to the high concentration of xanthophylls and fucoxanthines [14]. Various compounds with antibacterial, antifungal, and antiviral properties had been reported in marine macroalgae [15]. Macroalgae extracts are also widely used in agriculture as soil conditioners to improve crop yields [16,17,18]. Some authors have reported that these polysaccharides are rich in functional groups that can bind to trace elements that play an important nutritional role in plants [19]. The application of macroalgae to plants can improve their growth under conditions of frost, drought, and salinity, increase resistance to various diseases, and improve crop yield and productivity [20]. In particular, *Ascophyllum nodosum* (*Phaeophyceae*) has been extensively studied as a biofertilizer for application to crops and as a supplement for humans and animals [21]. Several biofertilizers have been produced using extracts of *Fucus serratus*, *A. nodosum*, and *Laminaria digitata* (*Phaeophyceae*), as they contain high levels of betaines, compounds that can protect plants against salinity, heat, and water stress [22]. Science is currently focused on producing plants using natural methods that are safe for the environment [23]. A secondary goal is to test marine macroalgae for their potential in agriculture, although most research focuses on in vitro screening [24,25]. The aim of this review was to highlight and summarize the potential of marine macroalgae and their possible derivatives that can be used in agricultural disease control, pest management, and improved crop production within the framework of sustainable management.

## 2. Historical and Economic Aspects of the Use of Macroalgae

Enthusiasm for using macroalgae in agriculture has grown significantly in recent decades, partly due to unusual proliferations of algal biomass, particularly green algae, resulting from the progressive eutrophication of certain coastal ecosystems. The impact of climate change exposes crops to more frequent biotic and abiotic stresses, leading to significant losses for farmers. Research is therefore focusing on developing sustainable methods to mitigate these stresses [26]. Several recent studies have shown that macroalgae extracts can protect plants from several biotic and abiotic stresses. Currently, the growing interest in environmentally sustainable cultivation practices, combined with the significant reduction in chemically synthesized pesticides permitted in both field and post-harvest applications, is stimulating the search for new easily available and cost-effective alternatives to conventional formulations [27]. Moreover, the side effects on soil and the environment caused by the excessive use of inorganic fertilizers are prompting scientists to consider alternative biofertilizers. The macroalgae most widely used in agriculture for their good biostimulating activity are the red algae *Ellisolandia elongata* (formerly *Corallina mediterranea*), *Jania rubens*, and *Pterocladia pinnata* (formerly *Pterocladia pinnata*), the green algae *Cladophora dalmatica* and *Ulva lactuca*, and the brown algae *Ascophyllum nodosum*, *Ecklonia maxima*, *Sargassum* spp., and *Macrocystis pyrifera* (*Phaeophyceae*) [28]. Already in prehistoric times, macroalgae were an important food and medicinal resource for humans [29]. The ancient Greeks used them as fertilizers [30] and the ancient Romans commonly used macroalgae as manure in their agricultural practices [31]. The first written reference to this use, dating back to the second half of the first century A.D., is by the Latin agronomist Lucius Junius Moderatus Columella, who recommended that cabbage roots be fertilized with algae. Furthermore, for centuries, in the coastal areas of the Atlantic Ocean (Brittany, Ireland), macroalgae, especially large brown ones, have traditionally been used to improve soil fertility. Agricultural areas near these coastal areas have always used macroalgae as an important source of organic matter to fertilize various types of soil and a variety of fruit and vegetable crops. For such purposes, pleustophytic or stranded seaweed is generally harvested, although in Scotland farmers sometimes cut the fronds of *A. nodosum* exposed by the low tide. In the UK, particularly in Cornwall, the most usual activity is to mix microalgae with sand and then bury them. Similar practices have been reported for Argentina as well, where large biomasses of green algae end up on the shore each summer, interfering with recreational use of beaches, and for the Philippines where large quantities of brown algae of the genus *Sargassum* are either collected and used fresh on a local scale or dried and transported to other areas [32]. Industrial production of algae preparations dates back to the 17th century [33], but the most significant advance in their agricultural use occurred during World War II, driven by studies seeking new sources of fiber. The first practical method to liquefy macroalgae for agricultural use was developed in 1949 [34]. In 1974, an Australian industry put the first organic liquid extract called ‘Seasol’ on the market [35]. Algae extracts thus represented the first biostimulants applied to plants to increase their productivity [36,37]. Today, algae-based soil conditioners are readily available in ready-to-use formulations for cultivated land and home gardens. The market offers a wide variety of high-quality products with different formulations (powder or liquid) and modes of administration, available in pure form or combined with other traditional ingredients such as fertilizers or pesticides [32]. Table 1 provides a compilation of various commercial seaweed products intended for agricultural and horticultural use, detailing their manufacturers and countries of origin.

In 2005, the world production of algae was 14.7 million tonnes, of which 13.5 million came from cultivation facilities, while in 2015, ten years later, production virtually doubled to 30.4 million tons, focusing especially on oceanic species [38]. Less than 1% of the total value of the current industry is still devoted to narrow agricultural use, while around half of the production is carried out as fodder. Despite this, interest in agricultural use of algae is undoubtedly growing, as evidenced by the exponential increase in scientific publications on the subject [34].

## 3. Macroalgae Fertilizers and Biostimulants

In agriculture, macroalgae are mainly used as biostimulants and biofertilizers to increase growth and improve plant yields. Macroalgae are widely used in the form of algal extracts or for direct application to the soil but are also used for compost production from fresh or dried and shredded thalli [39]. Farmers in more developed countries have begun to replace chemically synthesized plant protection products, used in conventional farming, with low-environmental-impact products suitable for organic and biodynamic alternative or sustainable farming [40]. To meet the growing demand for environmentally friendly fertilizers, the possibility of using macroalgae for their production has been explored. In contrast to chemical fertilizers, fertilizers derived from algae (mainly brown algae of the genera *Fucus* spp., *Laminaria* spp., *Ascophyllum* spp., and *Sargassum* spp.) are biodegradable and are not toxic, polluting, or dangerous to humans and animals as they do not bioaccumulate [41]. The acidity of the soil does not influence the application of biofertilizer algae; on the contrary, the application of algal formulations normally stabilizes the pH of the soil.

**Figure 1:**
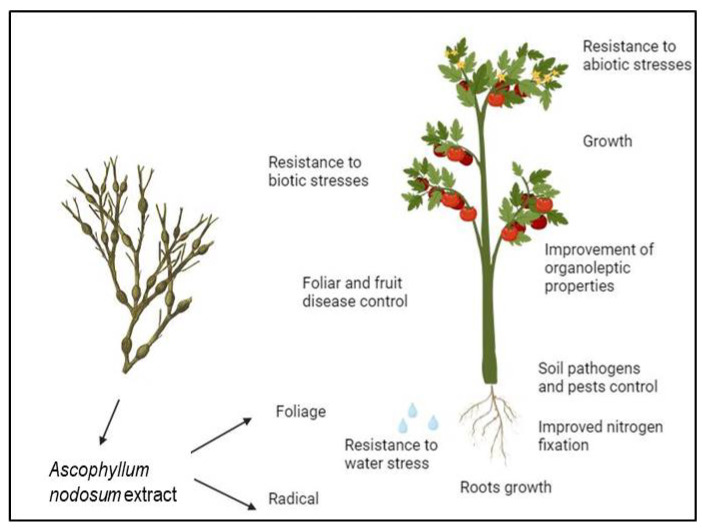
Schematic representation of the beneficial effects of algal extracts and possible mechanisms of bioactivity.

The beneficial effects of algal products used in agriculture (Figure 1) include the following:

- improved germination of seeds.
- improved plant yield.
- enhanced root growth.
- increased tolerance to various abiotic stresses.
- increased resistance to infection or insect attack.

These products contain essential nutrients for plants, ranging from trace elements to organic compounds such as carbohydrates, amino acids, and vitamins, as well as substances with a stimulating and antibiotic nature [42]. In recent years, techniques developed in the fields of molecular biology, metabolomics, and genomics have been used to evaluate the efficacy of these products and to learn about their mechanisms of action. With the complete or near-completion sequencing of several plant genomes, it is now possible to observe the effects of macroalgal extracts and components on the entire plant genome and transcriptome. This allows for a better understanding of the mechanisms behind algae-induced growth responses and stress reduction [26]. The use of algae as biofertilizers poses no risk to environmental biodiversity or eutrophication, as they are applied in lower dosages compared to conventional fertilizers and degrade quickly. These innovative tools do not directly contribute to plant nutrition, but when used appropriately, they can enhance crop yields by activating physiological and biochemical processes that improve resource efficiency, such as nutrient and water use. This reduces input requirements during the crop cycle and enhances crops’ resilience to abiotic stresses (thermal, saline, water, and nutrient deficiencies). In terms of agronomic efficacy, algal biostimulants are effective at concentrations significantly lower than fertilizers, but higher than growth regulators. While they support nutrient uptake, they cannot replace it.

### 3.1. Soil Improvers

The application of macroalgae as organic soil conditioners has increased in recent years due to growing awareness of the negative effects of chemical pesticides [43]. However, this practice has a long history, especially in coastal regions with sandy soils low in organic matter, where access to algae is more convenient [44]. One potential problem that could discourage its use is the salt content of seaweed macroalgae, although it is unlikely that growers would go so far as to add quantities that would seriously upset the salt balance in soils. In the case of repeated and direct applications of this type of compost, its electrical conductivity must be carefully controlled and monitored. To lower salinity, the algae material can be rinsed or desalinated by rainwater, or it can be watered frequently during composting. A negative effect of macroalgae rinsing is the risk of losing alginates, valuable components of brown algae in particular [45]. Alginic acid and its salts, called alginates, act as soil conditioning agents as they combine with metal ions to form polymers with a much higher molecular weight that swell, retain soil moisture, and improve soil structure, especially in the case of clay soils. These cross-linked polymers, in fact, optimize the aeration of the soil and the capillary activity of its pores, as well as the water retention characteristics of the soil itself [46]. As a result, root growth is stimulated, and soil microbial activity is improved [47,48,49]. However, distinguishing the direct effects on the physical properties of the soil from the indirect effects on soil microorganisms is challenging. Calcium alginates, in particular, form very strong gels, increasing the stability of soil aggregates [32]. In terms of structure, the volume does not increase significantly, but the gelatinous content of the alginates helps to bind the soil particles together [26,34]. Alginic acid, by sequestering aluminum and iron cations, can precipitate phosphates, thus increasing phosphorous availability [50]. Brown algae, of which the most common species belong to the genera *Ascophyllum*, *Ecklonia*, *Fucus*, and *Macrocystis* function both as fertilizers and soil conditioners as they have an adequate nitrogen and potassium content, but a lower phosphorus content than traditional animal fertilizers and the typical N:P:K ratio found in chemical fertilizers. In line with these results obtained with some brown algae, the red algae *Solieria robusta* was also found to be as effective a soil conditioner as chemical fertilizers, such as urea and potash, in improving the growth of soybean plants (*Glycine max* L.) [43]. Finally, another beneficial effect of algal products is exerted on root mycorrhizal symbioses. In particular, the application of red and green algae extracts to papaya (*Carica papaya* L.) and passion fruit (*Passiflora edulis*) roots resulted in the development of mycorrhizae to a greater extent than the control [51]. Similar results were obtained in a citrus grove treated with a liquid fertilizer containing the extract of *Laminaria japonica* (formerly *Laminaria japonica*) (*Phaeophyceae*) [52].

### 3.2. Algae Extracts and Liquid Fertilizers

**Figure 2:**
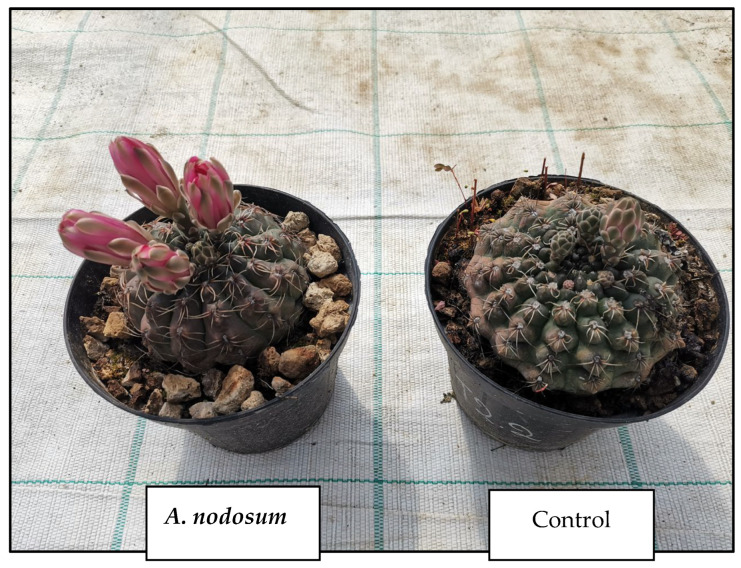
Effect of Ascophyllum nodosum on Gymnocalycium baldianum blooms.

These products are sold in concentrated form, making them easy to transport, applicable in appropriate dilutions, and quickly effective [32]. Foliar-sprayed fertilizers improve the nutrient uptake efficiency of the plants themselves. Nutrients do not leach from the soil but are accessed through leaf openings like lenticels, hydathodes, and stomata. The leaves generally absorb the nutrients within 10–15 min after application [53]. Foliar application of *A. nodosum* extract on grapevines (*Vitis vinifera* L.) after full flowering resulted in increased nutrient, anthocyanin, and phenol contents [54,55]. Two commercial extracts of the same algae, *A. nodosum*, enhanced the macronutrient and micronutrient content in tomato (*Lycopersicon esculentum* Mill.) fruit [54]. Similarly, olive (*Olea europaea* Mill.) plants showed increased uptake of potassium, iron, and copper [55]. When applied at 0.1% (*v*/*v*), the commercial extract of *A. nodosum* resulted in improved root and shoot growth of oilseed rape (*Brassica napus*) by stimulating nitrogen and sulfate accumulation [56]. Algae extracts are biodegradable and environmentally friendly, offering a promising alternative to synthetic plant stimulants whose application often leads to environmental pollution [57]. For these reasons, their use in organic farming has increased considerably. The extracts are marketed as liquid fertilizers and biostimulants because they contain numerous growth regulators like cytokinins [58,59], auxins [60], gibberellins [61], betaines [62,63], macronutrients such as calcium, phosphorus, and potassium, and micronutrients such as iron, copper, zinc, boron, manganese, cobalt, and molybdenum, which are necessary for plant development and growth [26]. In addition, many macroalgae, especially Phaeophyceae, have a high content of amino acids, antibiotics, and vitamins [64]. Commercial extracts are mainly produced from the brown algae *A. nodosum*, *Laminaria* spp., *E. maxima*, *Sargassum* spp., and *Durvillaea* spp., although other species such as *Fucus serratus (Phaeophyceae)*, *U. intestinalis*, *U. lactuca* (*Chlorophyta*), and *Kappaphycus alvarezii* (*Rhodophyta*) have also been used [34]. Some products are obtained by alkaline extraction and subsequent filtration; others consist of suspensions of very fine particles [32]. Biostimulants refer to natural substances that enhance vegetative growth, facilitate mineral nutrient absorption, and improve tolerance to both biotic and abiotic stresses [26]. Many algal extracts are bioactive already at low concentrations, at dilutions of 1:1000 or higher [42,65,66]. Macroalgae are a known source of growth regulators, organic osmolytes, amino acids, nutrients, vitamins, and vitamin precursors [67]. The main phytohormones identified in the extracts are auxins, cytokinins, gibberellins, abscisic acid, and ethylene [28]. In particular, auxins are responsible for plant tissue elongation and apical dominance, cell division, plant movement, and aging. Cytokinins are involved in the regulation of cell division affecting plant growth and resting period; they also inhibit plant tissue aging and play a crucial role in nutrient transport in both vegetative [68] and reproductive organs [69]. Algal extracts contribute to enhancing the mobilization of cytokinins from the roots to developing fruits. Alternatively, they may improve the quantity or synthesis of endogenous cytokinins within the fruits themselves, employing distinct mechanisms of action in monocotyledons and dicotyledons [70]. Some basic functions of gibberellins are the initiation of seed germination, growth regulation, interruption of bud dormancy, flowering, and fruit development. Abscisic acid and ethylene, on the other hand, are responsible for responding to stress factors, inhibiting cell growth, and accelerating aging. Finally, betaines, although not traditionally included among the classic plant hormones, have activity like cytokinins [67]. Betaines act as osmo-protectors to improve plant resistance to drought and salt stress [71,72]. Another observed effect, which has been attributed to the presence of betaines, is the increased chlorophyll content in the leaves of tomato, bean (*Phaseolus vulgaris* L.), barley (*Hordeum vulgare* L.), and maize (*Zea mays* L.) plants following the application of an *A. nodosum* extract [73]. The macroalgae also contain other interesting chemical compounds. For instance, a derivative of vitamin K1, known as kaidrin, enhances the efficiency of proton pumps and improves nutrient uptake by the roots. Polyamines such as putrescine and spermine can alleviate nutrient deficiency and accumulate in plants in response to stress [74]. Stimulating effects of algal extracts on vegetative growth have been reported in grapes, apple (*Malus domestica*), and watermelon (*Citrullus lanatus*) [75,76,77]. Other studies on pepper (*Capsicum annum* L.) seedlings have shown that repeated foliar applications of *A. nodosum* lead to an increase in fruit length, diameter, and yield [78]. Commonly, application of *A. nodosum* derivatives has resulted in increased yield and quantity in citrus fruits [79], grapes [80], apples [81], and pears [82]. The positive effect of foliar application of an extract obtained from *A. nodosum* on olive trees was also studied; the product resulted in an increase in olive size and improved quality and oil production per tree [83,84]. Positive effects of algal products on root growth and development were also observed as biostimulants, in general, improve lateral root formation [85,86], increasing the total volume of the root system [75,87,88]. Some algal extracts were also shown to be particularly active in controlling root-killing nematodes and pathogenic fungi responsible for post-harvest rot [89,90]. The application of algal concentrates can also reduce transplant shock in cabbage (*Brassica oleracea* L.) and tomato seedlings by increasing root size and vigor [42], as well as speed up the development of transplanted mango (*Mangifera indica* L.) seedlings [91]. With regard to the whole-plant response, it is worth mentioning that plants treated with *E. maxima* products showed an increase in the ratio of root to root dry mass, indicating that the algal components have a considerable effect on root growth [92]. In addition, algae concentrate can increase root size and, consequently, increase the volume of soil occupied by a plant, which becomes more efficient in the uptake of nutrients [67,76] and, in particular, those needed as components in protein synthesis (nitrogen, phosphorus, and sulfur), resulting in increased synthesis. Seedling emergence and enhanced vigor are widely recognized to significantly influence seedling establishment, growth, and development. Early emergence favors the establishment and rapid transformation of the plant from heterotrophic, a phase in which it relies on the reserve substances stored in the seeds, to autotrophic, with a functional photosynthetic apparatus [93]. A positive effect of the application of algal extracts on chlorophyll content has been suggested in various works, for example, in the case of the application of a low-concentration *A. nodosum* extract on tomato soil or foliage and on the flowering of *Gymnocalycium baldianum* (Figure 2). The increase in chlorophyll is the result of a reduction in the degradation of chlorophyll itself, partly due to the betaines present in the algal extract [94]. There is also a direct effect of improving the absorption of magnesium or iron, which is an essential element for chlorophyll biosynthesis [67,94]. Also, in another experiment on fenugreek plants (*Trigonella foenum-graecum* L.), the application of two liquid algal fertilizers produced by the species *Sargassum ilicifolium* (*Phaeophyceae*) and *Ulva lactuca* (formerly *Ulva fasciata*) (*Chlorophyta*) promoted the content of this pigment.

### 3.3. Tolerance to Abiotic Stresses

Reactive oxygen species (ROS) play a pivotal role in various abiotic stresses, including salinity, temperature extremes, and drought. The application of various algal products can alleviate such stresses. In detail, the treatment of turfgrasses with an extract of A. nodosum enhanced the activity of the antioxidant enzyme superoxide dismutase (SOD) [95]. Similarly, Ayad (1998) [96] reported increased activities of SOD, glutathione reductase (GR), and ascorbate peroxidase (APX) in fescue (*Festuca arundinacea*). When comparing different algal species in Korea, the total content of active phenolic compounds with antioxidant properties varied greatly from species to species, ranging between 3 and 126 mg/GAE per gram dry weight of *Hydroclathrus clathratus* and *Sargassum micracanthum* (*Phaeophyceae*), respectively [97]. Conversely, several studies propose that the beneficial anti-stress effects of algal extracts may be linked to their cytokinin activity. They mitigate stress-induced free radicals by directly eliminating them and preventing the formation of reactive oxygen species (ROS) [95,98]. According to other authors, bioactive chemicals other than cytokinins could be involved, involving both steric characteristics and aromatic ring substituents of polyphenolic compounds [99]. With regard to low-temperature stress, commercial formulations based on *A. nodosum* improved the freezing tolerance of grapes [75,100].

### 3.4. Effects on Plant Diseases and Pests

Plants have numerous adaptive defense mechanisms to counteract attacks by pathogens and insects. After sufficient induction, plants can develop an enhanced defense capability, often referred to as ‘induced resistance’ [101]; compounds involved in defense are often useful in protecting macroalgae from epiphytes [102]. Algal extracts have been reported to enhance resistance to pests and diseases [103]. According to Mercier et al. [104], two types of algal polysaccharides, laminarin and carrageenans, effectively trigger defense responses in tobacco (*Nicotiana tabacum* L.) leaves. These algal products not only influence plant physiology and metabolism but also enhance plant health by influencing the microbial community in the rhizosphere [26]. Today, given the concerns regarding the use of chemical fungicides in horticultural crops, alternative strategies utilizing non-synthetic products, such as those derived from algae, must be considered. It is widely recognized that among the various polysaccharides present in algal extracts, there are effective elicitors that enhance plant defense against diseases [105]. The defense mechanism relies on the perception of signal molecules known as elicitors or plant systemic inducers, capable of generating a physiological response in the plant; some resistance inducers act by simulating the presence of a pathogen or by producing molecules similar to the elicitors themselves. These compounds, also called biostimulants, include algal polysaccharides such as laminarin, fucoidan, and the alginates of brown algae such as *A. nodosum*, *Fucus vesiculosus*, and *Saccharina longicruris* (formerly *Saccharina longicruris*). Carrot plants (*Daucus carota* L.) sprayed with an algal extract of *A. nodosum* were shown to be less susceptible to alternariosis and gray mold caused by the genera *Alternaria* and *Botrytis*, respectively [106]. Similarly, alfalfa plants treated with extracts of green algae (*Ulva* spp.) showed increased resistance against *Colletotrichum trifolii* [107]. In addition, pepper plants treated with an extract of *A. nodosum* showed an accumulation of the phytoalexin capsidiol and increased peroxidase activity, which conferred better resistance to *Phytophthora capsici* attacks [108]. In another experiment, a significantly lower incidence of dollar spot caused by *Sclerotinia homeocarpa* was recorded in agrostis (*Agrostis stolonifera* L.), while Masny and collaborators (2004) [109] compared the antibotritic activity of two commercial liquid products on two strawberry (*Fragaria* L.) cultivars, but neither formulation reduced the incidence of gray mold (*Botrytis cinerea*) on the fruit. The application of algal extracts to plants can also result in a significant reduction in levels of soil-borne pathogenic fungi, the management of which is one of the greatest challenges facing modern agriculture on a global scale [39]. The primary necessity lies in finding alternatives to conventional strategies, such as relying solely on resistant cultivars or synthetic fungicides, as these methods often prove inadequate in controlling pathogens [110]. The proposed mode of action for algal products is linked to the presence of easily degradable organic matter in macroalgae, which is useful for feeding the proliferation of antagonistic bacteria, whose populations increase in the rhizosphere. An alternative hypothesis contemplates that algal alginates directly suppress pathogens [43]. Positive responses in this respect have been found to combat both rhizottoniosis in potatoes [111] and verticillosis in peppers [106] with the genera *Rhizoctonia* and *Verticillium* as etiological agents, respectively. In conclusion, algal extracts can be a useful supplement to reduce the input of conventional fungicides and fertilizers, while maintaining adequate plant health. Several works suggest that macroalgae contain elaborate secondary metabolites that play a significant role in host defense against predators, fungi, and parasites [112]. Previous studies have also revealed that sap-feeding insects generally avoid plants treated with algal extracts. Furthermore, red mite populations on strawberries were significantly reduced by bi-weekly spray treatments with a commercial product based on *A. nodosum* [113]. The mechanism of action remains unknown, but the extracts may contain chelated metals capable of reducing mite fertility [26]. Moreover, increased levels of anthocyanin and phenolic components in the leaves may alter their palatability for predatory insects. Losses caused by plant-parasitic nematodes are estimated to be around USD 100 billion per year [114]. Algal extracts increase plant resistance to nematodes, probably by altering the auxin/cytokinin ratio. Betaines from *A. nodosum* also caused a reduction in *Meloidogyne javanica* and *M. incognita* infestations in tomato [62]. In this study, macroalgae showed a suppressive effect on nematodes, quite similar to the chemical nematicide carbofuran. In other experiments, the use of *Soliera robusta* alone or in combination with fertilizers or pesticides significantly reduced nematode infection by reducing the formation of galls on soybean roots [43]. Finally, the antioxidant polyphenols present in macroalgae have bactericidal properties [115].

## 4. Algae Extraction Methods

In general, the extracts are obtained by processes using high-pressure water, alkalis or acids, alcohols, microwaves, CO_2_, or through mechanical breakdown by trituration at low temperature to obtain a micronized suspension of fine particles [116]. Alternatively, the algal cells are broken up using a high-pressure apparatus and the soluble cytosolic components are recovered in the filtered liquid [117]. The most commonly used process involves heating the algae with alkaline solutions of sodium and potassium. However, the use of alkali to liquefy the algal components can generate a number of reaction by-products not present in the starting material [34]. The nature and quantity of these compounds will depend on the composition and chemical structure of the polymers originally present in the algae, as well as the processing conditions used to produce the soluble extract. By means of microwave-assisted extraction combined with high-pressure water extraction, the polysaccharide fucoidan can be extracted. Cytokinins can be extracted using chilled ethanol at 70%, while extraction in methanol at 85% results in gibberellin-rich extracts.

## 5. How Seaweed Has Been Used in Agriculture

Seaweed is a resource that has been used for many years to fertilize soils dedicated to cultivation in places close to the coast. Nowadays, seaweed extracts and concentrates are produced and distributed to different places far from the coast. In the first case, fresh or dried seaweed (whole or crushed) takes months to be fully and effectively incorporated into the soil, as the nutrients must be broken down by bacteria before they can be used by the plants, whereas in extracts or concentrates the nutrients are separated and the effect is immediate [118]. Fresh, dehydrated, pulverized algae or extracts are products that are made from different species of algae that inhabit a certain area of interest where the resource is to be exploited. However, brown algae, due to the size and biomass they produce, are preferred for use by the agricultural industry, which faces challenges in meeting the demand for food for the population without damaging the environment. There are now studies showing why algae improve agricultural soil characteristics and crop production. Hashem et al. (2019) [119] point out that the application of algae as biofertilizers not only adds nutrients to the soil and plant for better growth and development, but also causes significant changes in plant metabolism for better adaptation to adverse environmental conditions. Likewise, Uribe-Orozco et al. (2018) [120] report that *Sargassum vulgare* meal is a strong plant rich in fiber, protein, carbohydrates, and lipids that contributed to improve the physicochemical properties of soil and increased the production of coriander crop. The different compounds contained in the seaweed contribute not only to improve soil characteristics and crop development, but also produce healthy and vigorous plants. Some recent research that applied seaweed products such as extracts and meal on different crops, reflecting improved plant development, is mentioned below. Hashem et al. (2019) [119] evaluated three macroalgae from different groups, *Ulva lactuca* Linnaeus (green algae), *Cystoseira* spp. (brown algae), and *Gelidium crinale* (Hare ex Turner) *Gaillon* (red algae), as a soil amendment to improve the growth and yield of *Brassica napus* L. (canola) under normal conditions and under salt stress (NaCl 75 and 150 mM). All three applied algae showed positive effects under both normal and salt stress conditions compared to untreated plants. However, the most effective treatment was with *U. lactuca* because it contained significantly higher levels of total carbohydrates, glycerol, proline, antioxidant activity, and phytohormones such as AIA (indole acetic acid), zeatin, and benzyl adenine compared to the other algae tested. The highest levels of AIB (indole butyric acid) and ABA (abscisic acid) were detected in G. crinale, while GA3 (gibberellic acid) and AJ (jasmonic acid) were found in *Cystoseira* spp. Uribe-Orozco et al. (2018) [120] analyzed the effect of the brown alga *Sargassum vulgare* C. Agardh in soil and on the growth of coriander plants, which was evaluated with the length and total dry biomass at 90 days. It was observed that the application of 6 and 9 g of flour provided more nutrients to the soil and consequently the plant developed better. In addition, changes in pH and EC (electrical conductivity) were determined after 35 days of cultivation. Seaweeds are also used for composting different organic wastes. Lacatusu et al. (2017) [121] evaluated compost with three organic wastes: 50% seaweed of *Cladophora* sp. and *Ulva lactuca* Linnaeus species, equal amounts of 25% farmyard manure and residual sludge. The soil presented favorable conditions for the cultivation and development of maize plants and at the end of the experiment the compost-treated soil contained more nutrients, higher humidity, improved air circulation, and an environment for the activity of microorganisms compared to the control and even to the mineral fertilizer treatment. The authors conclude that composting is a useful low-cost technology that allows transforming organic waste into a stable product such as organic fertilizer. In the research by Michalak and Chojnacka (2015) [122], it is mentioned that seaweeds are used as fertilizers and biostimulant compounds that are obtained through water extraction processes (autoclave), where the different compounds are released from the seaweed biomass. These are applied to the soil, to the seeds before sowing, and to the foliar part of the cultivated plants, increasing crop production and improving soil characteristics. Furthermore, Blunden et al. (2010) [123] evaluated methanolic extracts of *Ascophyllum nodosum* (Linnaeus) Le Jolis, *Laminaria digitata* (Hudson) J.V. Lamouroux, *Laminaria hyperborea* (Gennerus) *Foslie*, and *Fucus serratus* L., with which biofertilizer is produced due to their high content of betaines, organic osmolytic compounds that can play a crucial role in effective protection against salts, drought, and extreme temperature stress.

## 6. Other Applications of Algal Extracts

In addition to the established use of algal extracts in field crops, there are other specialized applications where extracts can be effectively used to promote growth, e.g., in hydroponic systems or in in vitro tissue culture. Weekly application of extracts in hydroponic solution resulted in significant increases in growth rate and other parameters in barley seedlings compared to the control [124]. Also, in potato (*Solanum tuberosum* L.) seedlings grown in vitro, an elongation of shoots and better development of lateral buds, as well as tuberization, occurred when algal extracts were applied to the culture medium. The improvement in seedling quality and rooting capacity also resulted in better establishment in the greenhouse [124].

## 7. Conclusions

The use of macroalgae and their extracts in agriculture is becoming increasingly popular worldwide. Further research into their biochemical nature and mechanisms of action may enable them to be fully exploited for agricultural production. Algal extracts are known to protect plants from a variety of biotic and abiotic stresses and offer high potential for application in the field and beyond. Currently, the most widely used species are large brown algae, but especially in some areas, such as the Mediterranean basin where they are generally not present, it would be desirable to identify new sources of extracts from macroalgae species that are easy to collect and find. The use of algal extracts in agriculture not only reduces the application of harmful chemicals, but also helps to protect the environment. Their integration into agricultural practices around the world can sustainably increase crop yields, also in view of the projected population increase on a global scale by 2050. This will be one of the major challenges of the new millennium and researchers and all stakeholders must be ready to seize it.

## Figures and Tables

**Table 1:** Some commercial macroalgae products for agricultural use.

Trade Name	Macroalgae	Producer and Country of Origin	Application
Akadian Plant Health^TM^	*Ascophyllum nodosum*	Acadian Seaplants —Canada	Biostimulant
Algalis	*A. nodosum*	LG—Italy	Biostimulant
AgroKelp	*Ascophyllum nodosum*	Algas y Biod. S.A.—Mexico	Biostimulant—Fertilizer
Alga Special	*A. nodosum*	L. Gobbi srl—Italy	Fertilizer
AlgaMaxima	*Ecklonia maxima*	C.R.A. srl—Italy	Biostimulant
Algaenzims	*Sargassum* spp.	Palau Bioquim—Mexico	Biostimulant
AlgaPlus FL	Brown Algae	Icas—Italy	Fertilizer
Algaroot	*Sargassum* spp.	Palau Bioquim—Mexico	Radicant
Biovita	*A. nodosum*	PI Industries Ltd.—India	Biostimulant
Cremalga	*A. nodosum, E. maxima, Macrocystis pyrifera*	Biolchim SPA—Italy	Biostimulant
Espoma	*A. nodosum*	The Espoma Company—USA	Biostimulant
Fylloton	Brown algae	Biolchim SPA—Italy	Biostimulant—Fertilizer
Guarantee	*A. nodosum*	Ocean Organics—New Zealand	Biostimulant
Kelp Meal	*A. nodosum*	Acadian Seaplants Ltd.—Canada	Biostimulant
Kelpak	*E. maxima*	BASF—Germany	Biostimulant
Kelprosoil	*M. pyrifera*	Productos del Pacifico	Biostimulant
Laminex	Brown algae	LG—Italy	Biostimulant
Mc Cream	*A. nodosum*	Valagro—Italy	Biostimulant
Micronalga	*A nodosum*	Biolchim SPA—Italy	Biostimulant
Seasol	*Durvillaea potatorum*	Seasol International—Australia	Biostimulant
Seaweed	*M. pyrifera*	Algas Marinas—Mexico	Biostimulant
Radicifo L 24	*Macrocystis pyrifera,* Zinc	Cifo srl—Italy	Biostimulant
Stimplex	*A. nodosum*	Acadian Agritech—Canada	Biostimulant
Turboenzims	*Sargassum* spp.	Palau Bioquim—Mexico	Bioinducer

## Data Availability

All data, tables, and figures in this manuscript are original.
